# Local electromechanical alterations determine the left ventricle rotational dynamics in CRT-eligible heart failure patients

**DOI:** 10.1038/s41598-021-82793-1

**Published:** 2021-02-05

**Authors:** Tomasz Jadczyk, Radoslaw Kurzelowski, Krzysztof S. Golba, Jacek Wilczek, Guido Caluori, Francesco Maffessanti, Jolanta Biernat, Katarzyna Gruszczynska, Magdalena Cybulska, Maximilian Y. Emmert, Zofia Parma, Kamil Baranski, Mieczyslaw Dutka, Barbara Kalanska-Lukasik, Zdenek Starek, Wojciech Wojakowski

**Affiliations:** 1 Department of Cardiology and Structural Heart Disease, Medical University of Silesia, Ziołowa 45-47, Katowice, Poland; 2 Interventional Cardiac Electrophysiology Group, International Clinical Research Center, St. Anne’s University Hospital in Brno, Brno, Czech Republic; 3 Department of Electrocardiology and Heart Failure, Medical University of Silesia, Katowice, Poland; 4 IHU-LIRYC, Inserm U1045 (CRBCT), Bordeaux, France; 5 Center for Computational Medicine in Cardiology, Università Della Svizzera Italiana, Lugano, Switzerland; 6 Department of Diagnostic Imaging, Medical University of Silesia, Katowice, Poland; 7 Department of Cardiovascular Surgery, Charité Universitätsmedizin Berlin, Berlin, Germany; 8 Department of Cardiothoracic and Vascular Surgery, German Heart Center Berlin, Berlin, Germany; 9 Department of Epidemiology, Medical University of Silesia, Katowice, Poland; 10 Department of Biochemistry and Molecular Biology, Faculty of Health Sciences, University of Bielsko-Biala, Bielsko-Biała, Poland; 11 1st Department of Internal Medicine-Cardioangiology, St. Anne’s University Hospital in Brno, Brno, Czech Republic; 12 Institute for Regenerative Medicine (IREM), University of Zurich, Zurich, Switzerland

**Keywords:** Heart failure, Cardiology

## Abstract

Left ventricle, LV wringing wall motion relies on physiological muscle fiber orientation, fibrotic status, and electromechanics (EM). The loss of proper EM activation can lead to rigid-body-type (RBT) LV rotation, which is associated with advanced heart failure (HF) and challenges in resynchronization. To describe the EM coupling and scar tissue burden with respect to rotational patterns observed on the LV in patients with ischemic heart failure with reduced ejection fraction (HFrEF) left bundle branch block (LBBB). Thirty patients with HFrEF/LBBB underwent EM analysis of the left ventricle using an invasive electro-mechanical catheter mapping system (NOGA XP, Biosense Webster). The following parameters were evaluated: rotation angle; rotation velocity; unipolar/bipolar voltage; local activation time, LAT; local electro-mechanical delay, LEMD; total electro-mechanical delay, TEMD. Patients underwent late-gadolinium enhancement cMRI when possible. The different LV rotation pattern served as sole parameter for patients’ grouping into two categories: wringing rotation (Group A, n = 6) and RBT rotation (Group B, n = 24). All parameters were aggregated into a nine segment, three sector and whole LV models, and compared at multiple scales. Segmental statistical analysis in Group B revealed significant inhomogeneities, across the LV, regarding voltage level, scar burdening, and LEMD changes: correlation analysis showed correspondently a loss of synchronization between electrical (LAT) and mechanical activation (TEMD). On contrary, Group A (relatively low number of patients) did not present significant differences in LEMD across LV segments, therefore electrical (LAT) and mechanical (TEMD) activation were well synchronized. Fibrosis burden was in general associated with areas of low voltage. The rotational behavior of LV in HF/LBBB patients is determined by the local alteration of EM coupling. These findings serve as a strong basic groundwork for a hypothesis that EM analysis may predict CRT response.

**Clinical trial registration:** SUM No. KNW/0022/KB1/17/15.

## Introduction

The physiological wringing movement of the left ventricle (LV) is determined by several anatomical and physiological features^1^.

The electromechanical (EM) activation pattern is not transmurally homogenous because of the subendocardial location of the His-Purkinje system and the anisotropic conduction along muscle fibers^2^. Furthermore, muscle fibers are organized in a three-dimensional (3D) counter-directional orientation across the myocardium (right-handed in the subendocardium and left-handed in subepicardium). These factors cause a endocardial-epicardial activation, followed by an endocardial isovolumetric shortening and subsequent epicardial shortening/twisting. Consequently, during systole, LV base and apex rotate in opposite directions—clockwise and counterclockwise, respectively. The synchrony between electrical and mechanical activation patterns is influenced by the presence of fibrosis and the tissue EM coupling.

In patients with heart failure (HF) and co-existing left bundle branch block (LBBB), the abnormal electrical activation, combined with post-ischemic EM coupling remodeling and geometrical alterations, may lead to dyssynchronous mechanical activation^3,4^. This can manifest in reduction of LV ejection fraction (LVEF), as well as changes in LV rotational pattern^5^. Rigid body-type (RBT) rotation—with basal and apical segments rotating in the same direction—was described by Setser et al. in patients with end-stage HF^6^. These findings were previously reported with the support of cardiac magnetic resonance tagging and speckle tracking echocardiography^7–11^. High RBT prevalence (up to 76%) was observed among the HF population with ischemic and non-ischemic etiology^6^.

Patients with ventricular dyssynchrony are eligible for the implantation of resynchronization devices that provide cardiac resynchronization therapy (CRT). CRT can restore LVEF, LV activation and pre-remodeling status, but 35–40% of patients do not respond to the therapy. Non-response to CRT is multifactorial, primarily depending on the site of pacing^12^, which can be optimized based on the LV patient-specific dynamics. The analysis of the LV electromechanical activation and torsion is increasingly gaining interest over the last years as a potential new diagnostic parameter of cardiac dysfunction and CRT optimization in HF patients^13^. Nevertheless, it is not clear to what extent the fibrotic burden or EM coupling alterations contribute to the LV rotation drastic alterations, which, in case of RBT rotation, could provide challenging to restore via CRT.

In the presented study, we describe the EM coupling and scar tissue burden with respect to rotational patterns observed on the LV in patients with ischemic heart failure with reduced ejection fraction (HFrEF) and LBBB, referred to CRT. We elucidate from the multiscale and multimodal comparative analysis which parameters and their interplay are significantly altered in presence of different LV rotation kinetics.

## Results

### Patients characteristics

**Figure 1:**
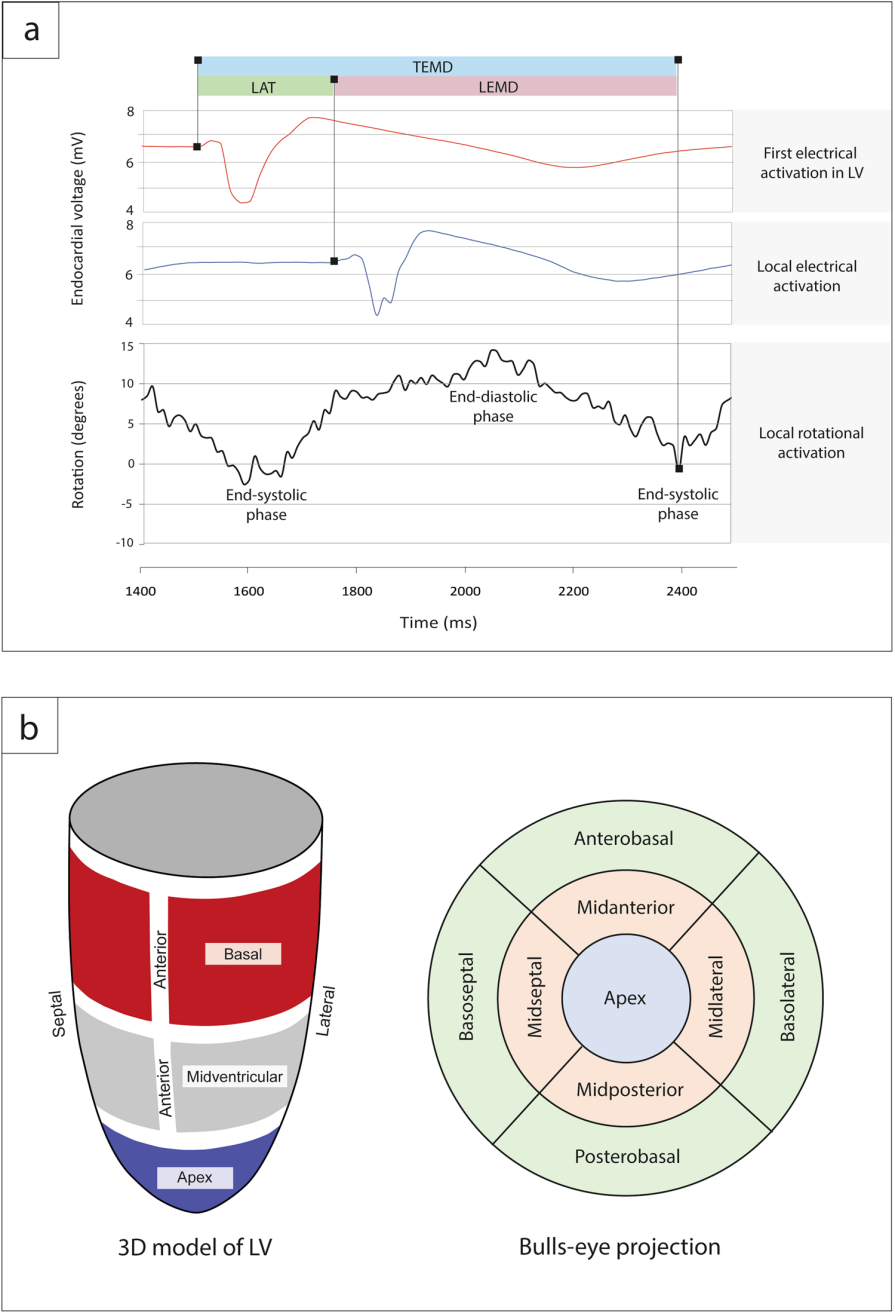
(**a**) Analyzed electro-mechanical parameters. (**b**) Standardized 9-segment bulls-eye projection in NOGA XP system.

Patient baseline characteristics is presented in Table 1. Study population mean age was 65.4 ± 6.1 years with a numerically higher number of males among all participants (70%). All patients had LBBB morphology on the ECG with mean QRS duration 168 ± 17 ms. Using the 3D electro-mechanical NOGA XP system, a total of 8200 endocardial mapping points was evaluated in the 30 HFrEF patients (273 ± 47 mapping points per patient, around 30 min per patient) and EM parameters were calculated (Fig. 1a,b and “Methods”).

**Table 1:** Baseline patient characteristics.

Parameter	Wringing rotation Group A	Rigid-body-type rotation Group B	p value
Age (years)	70.5 ± 3.8	64.2 ± 5.9	0.02
Male, n (%)	3 (50)	18 (75)	0.30
**ECG characteristics**			
QRS duration (ms)	170 ± 29	168 ± 16	0.80
LBBB morphology, n (%)	6 (100)	24 (100)	1.0
NYHA functional class			
II, n (%)	4 (66.7)	12 (50)	0.37
III, n (%)	2 (33.3)	12 (54.5)	0.37
**HF etiology**			
Ischemic, n (%)	6 (100)	24 (100)	1.0
**Medications, n (%)**			
ACE-Is/ARBs	6 (100)	24 (100)	1.0
Beta-blockers	6 (100)	24 (100)	1.0
Diuretics and/or spironolactone	6 (100)	23 (95.8)	1.0
LVEDV (ml)	247.0 ± 106.8	238.9 ± 54.4	0.79
LVESV (ml)	181.5 ± 80.8	177.6 ± 40.8	0.87
LVEF (%)	27.0 ± 10.0	25.7 ± 5.0	0.65
Sphericity index	1.65 ± 0.13	1.59 ± 0.16	0.42

Values are mean ± SD, median (25th; 75th percentile) or n (%).

*ACE-I* angiotensin-converting-enzyme inhibitor, *ARB* angiotensin II receptor blocker, *LVEDV* left ventricle end-diastolic volume, *LVESV* left ventricle end-systolic volume, *LVEF* left ventricle ejection fraction.

**Figure 2:**
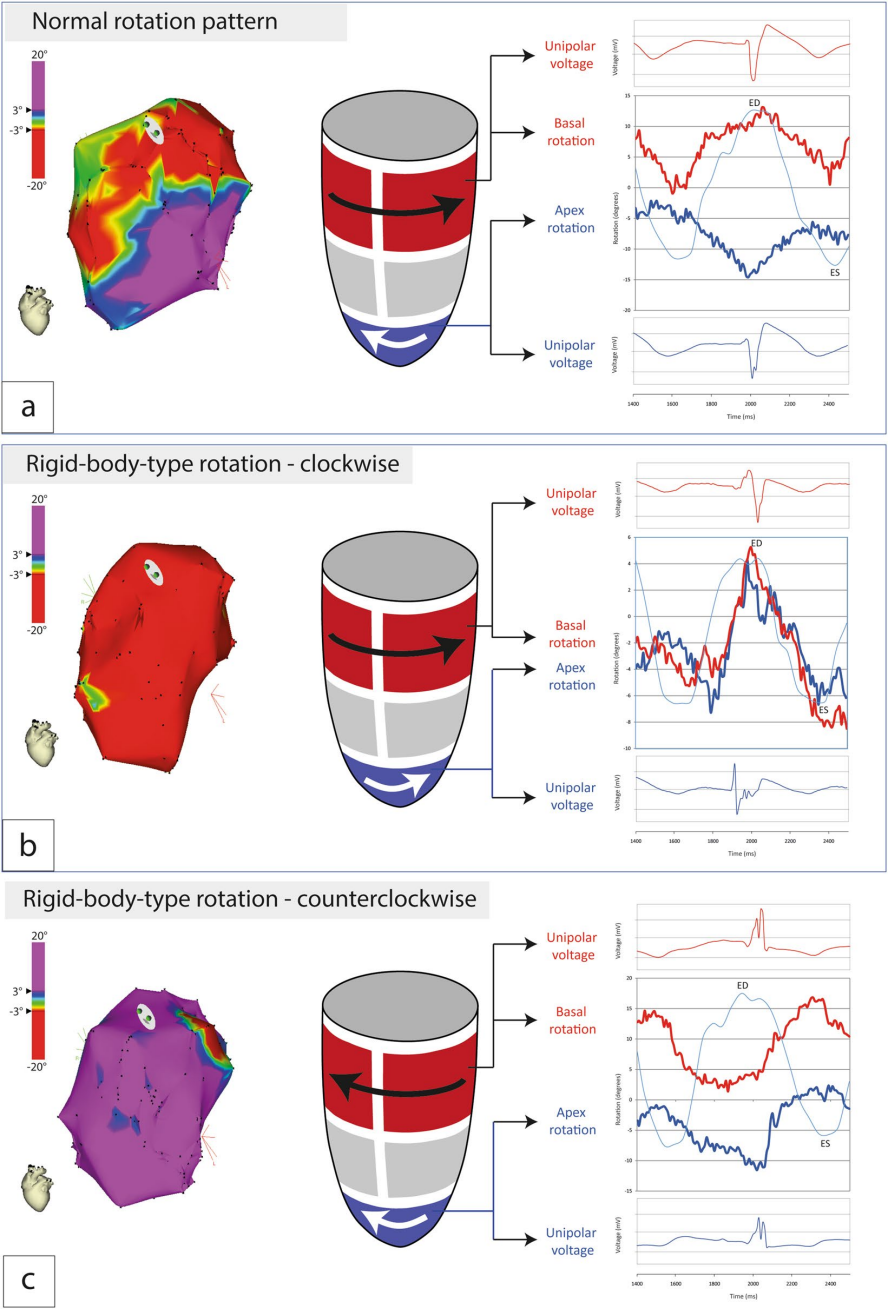
Left ventricle rotation patterns in patients with HFrEF and LBBB. (**a**) Apex–basal mechanical and electrical function in patient with wringing rotation pattern (characteristic for healthy individuals and observed in a subgroup of LBBB patients). (**b**) Apex–basal mechanical and electrical function in patient with predominantly clockwise rotation pattern. (**c**) Apex–basal mechanical and electrical function in patient with predominantly counterclockwise rotation pattern. Red areas indicate clockwise rotation (> 3°); Purple areas indicate counterclockwise rotation (< -3°).

Based on quantitative (clockwise [< − 3°]/counterclockwise [> 3°]) and qualitative (apex-base relative rotation) analysis of LV rotation at each endocardial mapping point, patients were classified as: wringing rotation (Group A, Fig. 2a, Supplementary Fig. S2A, Supplementary Video S1); rigid body-type rotation (Group B), clockwise (Fig. 2b, Supplementary Fig. S2b, Supplementary Video S2) or counterclockwise (Fig. 2c, Supplementary Fig. S2c, Supplementary Video S3).

With these criteria, 20% of patients were classified in Group A (n = 6), while 80% in Group B (n = 24). Group B showed predominantly clockwise rigid body-type rotation in 73% of patients (n = 22), and predominantly counterclockwise rigid body-type rotation in 7% of individuals (n = 2). Importantly, there were no statistically significant differences between groups in NYHA functional class (53% had NYHA class II, 47% NYHA class III). Mean LVEF was 26 ± 5%, mean LVEDV and LVESV were 240.5 ± 52.4 ml and 178.4 ± 41.2 ml, respectively. LV sphericity index (long-to-short LV axis ratio) was 1.65 ± 0.13 vs. 1.59 ± 0.16 for Group A and Group B, respectively, p = 0.42.

### Global rotational and electro-mechanical parameters

Global rotational and EM parameters values and group comparisons are detailed in Table 2.

**Table 2:** Global rotational and electro-mechanical parameters of LV.

Parameter	Wringing rotation Group A	Rigid-body-type rotation Group B	p value
LV peak torsion (°)	6.2 ± 3.0	3.5 ± 2.2	0.02
LV mean torsion (°)	4.3 ± 2.5	1.4 ± 1.6	0.002
LV rotation rate (°/s)	29.7 ± 9.2	40.0 ± 12.3	0.07
Interventricular delay (ms)	23 (10; 60)	51 (0; 79)	0.75
LV electrical activation time (ms)	120 ± 261	110 ± 22	0.37
LV rotational electro-mechanical delay (ms)	369 ± 59	366 ± 54	0.70
LV electrical cycle length (ms)	943 ± 106	869 ± 128	0.20
LV mechanical cycle length (ms)	950 ± 100	881 ± 123	0.22
Unipolar voltage (mV)	7.86 ± 3.01	9.84 ± 4.21	0.004
Bipolar voltage (mV)	1.55 ± 2.76	2.34 ± 2.49	0.007
LGE intensity (%)	13.87 ± 17.21	11.19 ± 17.64	0.225
Total annotation points number, n	272 ± 41	274 ± 50	0.93

Values are mean ± SD or median (25th; 75th percentile).

*LGE* late gadolinium enhancement, *LV* left ventricle.

#### LV rotational properties

Across the LV, patients’ groups showed significant differences in LV peak and mean torsion (4.3 ± 2.5° vs. 1.4 ± 1.6° for Group A and Groups B respectively, p = 0.002). No significant difference was found regarding the rotation rate (29.7 ± 9.2 m/s vs. 40.0 ± 12.3 m/s for Group A and Groups B, respectively, p = 0.07).

#### LV EM characteristics

No difference was observed between groups in terms of interventricular delay, LV electrical activation time (120 ± 261 ms vs. 110 ± 22 ms for Group A and Groups B respectively, p = 0.367), electro-mechanical delay (369 ± 59 ms vs. 366 ± 54 ms, p = 0.703), electrical/mechanical cycle length, and fibrosis burden (13.87 ± 17.21% vs 11.19 ± 17.64%, p = 0.225). LGE signal was higher at the apico-septal region, with varying involvement of the anterior or posterior wall. A significant difference was observed for unipolar (7.86 ± 3.01 mV vs. 9.84 ± 4.21 mV, p = 0.004) and bipolar voltage (1.55 ± 2.76 mV vs. 2.34 ± 2.49 mV, p = 0.007).

This evidence suggests that, at the global level, the groups present similar electrical activation times, EM coupling, contraction dynamics, and fibrosis burden, albeit with increased voltage amplitudes for Group B.

### Sectorial rotational and electro-mechanical parameters

Sectorial rotational and EM parameters values and group comparisons are detailed in Supplementary Tables S2 and S3. Notable findings are described in the following paragraphs.

#### LV sectors rotational properties

**Figure 3:**
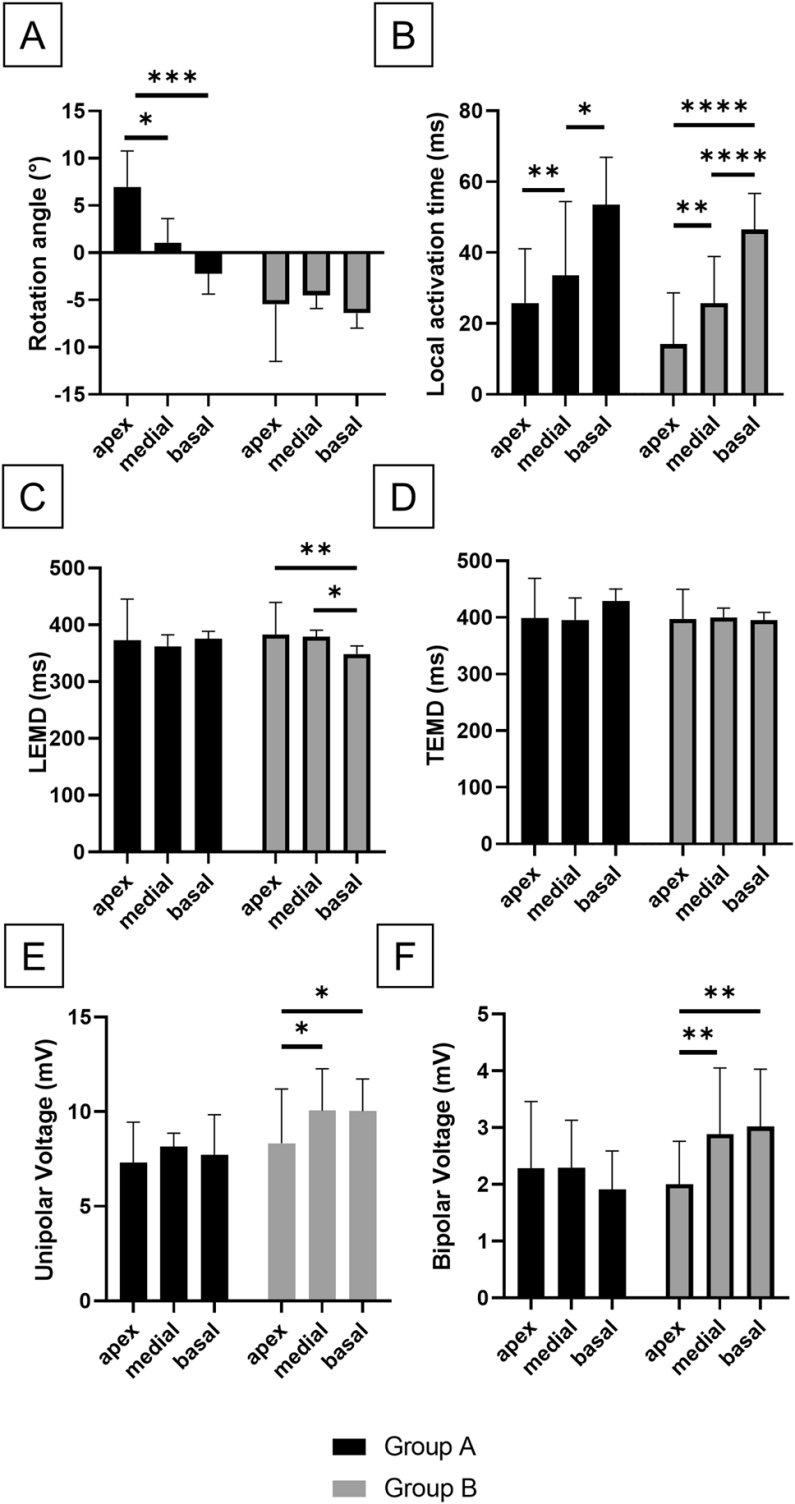
Graphical representation of electro-mechanical parameters statistical comparisons. (**A**) Rotational angle. (**B**) Local activation time. (**C**) LEMD. (**D**) TEMD. (**E**) Unipolar voltage. (**F**) Bipolar voltage. LEMD, Local electromechanical delay. *TEMD* total electromechanical delay. *p < 0.05; **p < 0.01; ***p < 0.001; ****p < 0.0001.

Intergroup comparisons between the two groups showed significant difference between all the sectors (Supplementary Table S2). Group B showed no change in average rotation angle across the apex, medial and basal sector. Due to the wringing motion in Group A, significant differences were observed between apical vs. medial sector (p = 0.016), and apical vs. basal ones (p < 0.0001) (Fig. 3a, Supplementary Table S3).

As per rotation velocity, intergroup comparisons showed significant differences at the medial sector (p = 0.003, Supplementary Table S2). Group B did not show intragroup sectorial differences, whilst Group A showed significant difference between apex vs. medial sectors (p = 0.001) and apex vs. basal ones (p = 0.042).

From this analysis results that Group A kinetics are dominated by the apex and the base in opposition, with medial sector as saddle point; group B kinetics appear homogenous across the sectors.

#### LV sectors EM characteristics

No difference was observed across Group A in terms of unipolar or bipolar voltage. Conversely, Group B showed significantly reduced voltage levels in the apex with respect to the medial and basal sector (Fig. 3E,F, Supplementary Table S3). Group B also presented higher basal bipolar voltage with respect to Group A (1.91 ± 0.68 mV vs. 3.02 ± 1.01 mV respectively, p = 0.044, Supplementary Table S2).

Examining the activation time, in both groups we observed a physiological and significant difference between the activation time of the different sectors: the activation time increased from the apex to the base, in the intragroup analysis (Fig. 3B, Supplementary Table S3). No difference was observed between the groups (Supplementary Table S2).

In the analysis of the LEMD, Group A did not present significant differences across sectors, but Group B showed statistically significant differences in LEMD between the apex and the basal/medial sectors (p = 0.004 and = 0.015 respectively, Fig. 3C). No difference was found when comparing sectors or segments of Group A vs. Group B.

TEMD was not significantly different across the sectors within Group A and Group B or between the two groups (Fig. 3D).

There was no difference in the fibrosis burden measured by LGE between the two groups’ sectors. At the same time, both groups presented internally significant differences between the apex (more fibrotic) and the medial/basal sectors.

The findings at the sectorial level are presented in the Box 1.

**Box 1: Summary of findings at the sectorial level** Group B shows basal and medial higher voltage.

The electrical activation pattern quantified by LAT is physiological for both groups.

In Group B the apex presents a faster EM coupling in the medial and basal sectors.

Mechanical activation is similar in and between both groups.

Scar burden was preferentially apical in both groups, with no differences between.

### Segmental rotational and electro-mechanical parameters

Segmental rotational and EM parameters values and group comparisons are detailed in Supplementary Tables S4 and S5. Notable findings are described in the following paragraphs.

#### LV segments rotational properties

Intergroup comparisons showed significant difference between the apex and midlateral segment (p < 0.0001 and p = 0.007 respectively, Supplementary Table S4). Group B showed no change in average rotation angle across the LV segments. Regarding Group A, intragroup comparisons showed significant differences in half of the segments (Supplementary Table S5).

Regarding the rotation velocity, the intergroup comparisons showed significant differences between the midlateral segment (p = 0.041, Supplementary Table S4). Group B did not show intragroup segmental differences, whilst Group A showed significant difference between the apex and the posterior segments.

From this analysis results that Group B kinetics are comparable across all segments, whilst Group A is characterized by opposite rotation with the base—including the whole anterior wall—and lower velocity in the posterior wall, when compared to the apex.

#### LV segments EM characteristics

**Figure 4:**
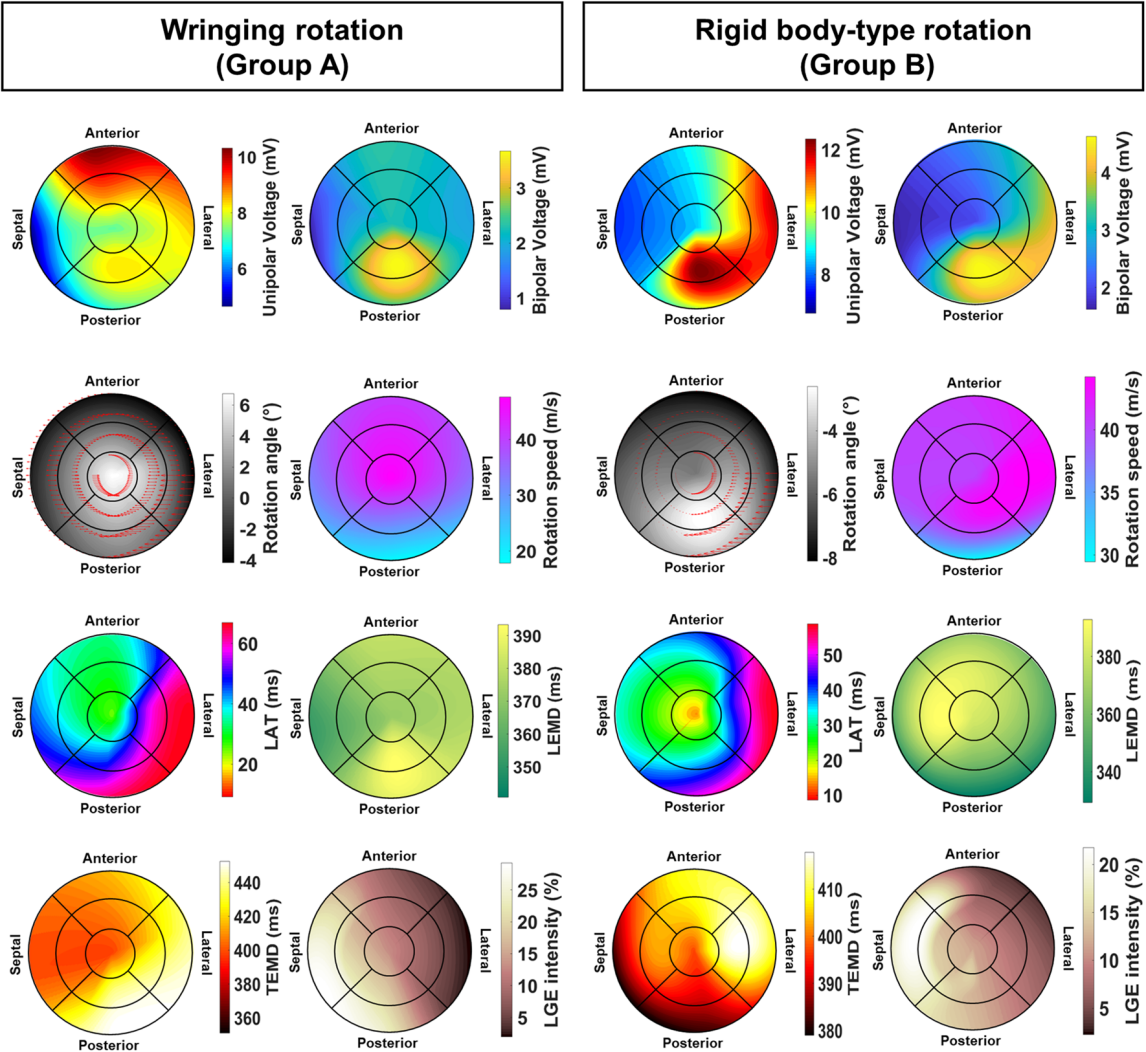
High-resolution aggregated maps of parameters of interest for the kinematic groups individuated, LV wringing rotation (Group A) and LV rigid-body-type rotation (Group B). Maps are oversampled by a factor 100 from the original nine segments with a spline function. *LAT* local activation time, *LEMD* local electro-mechanical delay, *TEMD* total electro-mechanical delay.

There was no significant difference in Group A concerning unipolar or bipolar voltage (Fig. 4a, Supplementary Table S5). For Group B, on the contrary, we found several significant differences between segments, specifically between the apex and the posterior and basolateral segments (Fig. 4b, Supplementary Table S5). No difference was found in the segmental analysis between different groups for unipolar or bipolar voltage, except for a higher unipolar voltage in Group B in the midposterior segment (8.39 ± 3.92 mV vs 12.45 ± 4.68 mV, p = 0.023, Supplementary Table S4).

**Figure 5:**
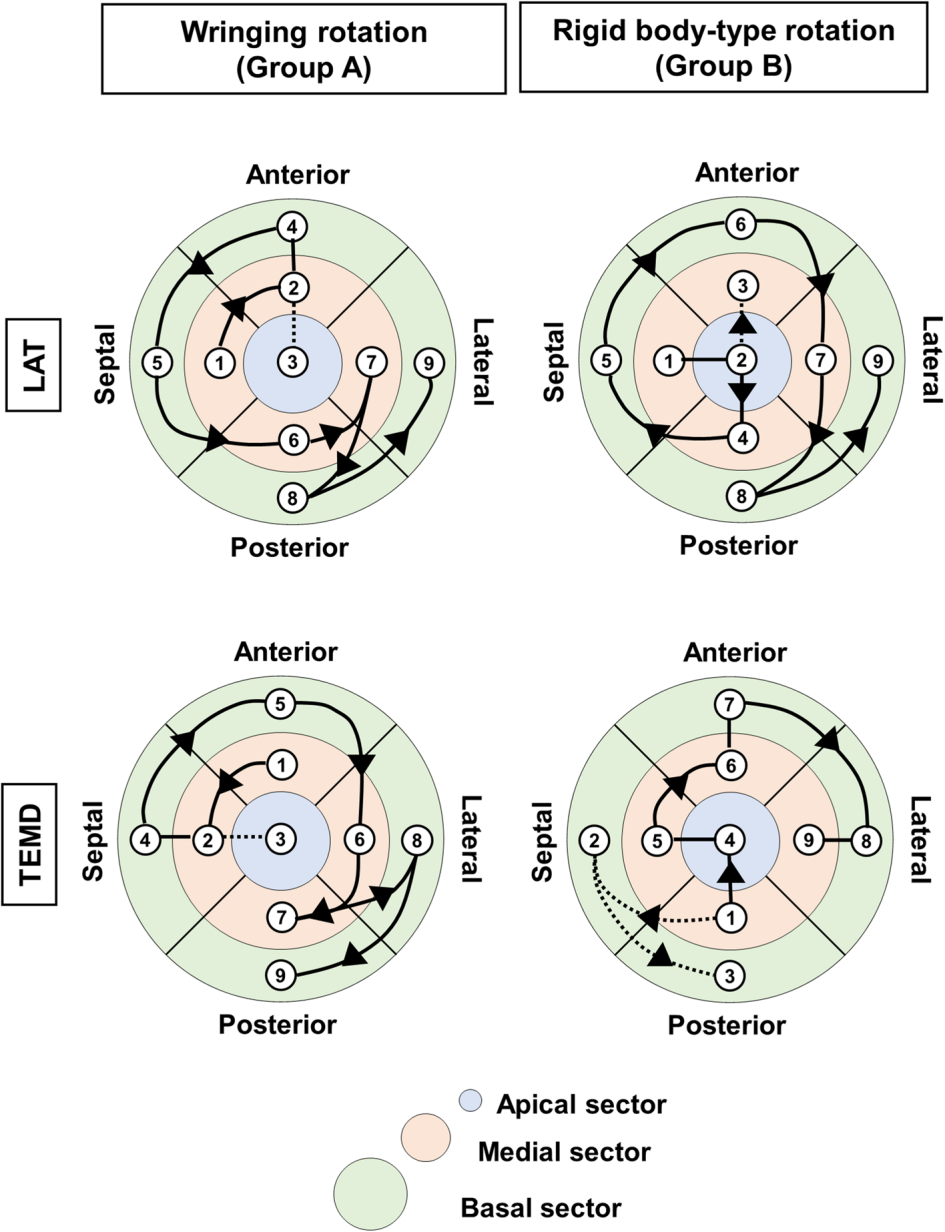
Segmental analysis of electro-mechanical activation fronts. Local activation time (LAT, top row), and total electro-mechanical delay (TEMD, bottom row) for the groups of left ventricle motion individuated. Numbers show the succession of segmental activation, connected by lines and arrows. Lines change dashing when a breakpoint on the electro-mechanical propagation front is observed.

In the analysis of the segmental activation time, we observed that in both groups, the midseptal segment was activated earlier for both groups, at local activation time (LAT) of 12 ± 19 ms and 10 ± 11 ms, respectively (p > 1.000); basolateral segments were activated late at LAT 67 ± 23 ms and 60 ± 17 ms, respectively (p = 0.986). Electrical activation front in both groups travels from the midseptal region to the basolateral one (Fig. 5a), yet in opposite directions, suggesting a shifted LAT conduction pattern.

Group A did not present significant differences in LEMD across its segments. In contrast, Group B showed statistically significant differences in LEMD between the apical vs. posterobasal ones. No difference was found when comparing segments of Group A vs. Group B. Nevertheless, we observed that in Group A the postero-lateral side is the one that presents the longer LEMD; in Group B the longest LEMD is found in the medial anteroseptal segments and in the apex.

TEMD segmental analysis between groups showed lower posterobasal TEMD in Group B (454 ms vs. 382 ms, p = 0.045), and no difference in the intragroup segmental analysis. In Group A, TEMD propagation shows that contraction originates from the mid-anterior segment, which travels to the apex and then spreads in a single front—counterdirectional from the LAT pattern—from the septal-anterior segments to the latero-posterior ones (Fig. 5B left); in Group B, the front slits in two with the septal and posterior segments activating before the apex and the anterolateral ones (Fig. 5B right).

LGE intensity segmental analysis showed no difference between the two groups. In the intragroup analysis, Group B presented a significantly more burdened medioseptal segment with respect to all the other segments, except the apex and the basoseptal ones; no difference was observed across Group A. Box 2 highlights the segmental EM analysis findings.

**Box 2: Summary of findings at the segmental level** Unipolar and bipolar voltages are inhomogeneous across the segments for Group B.

Electrical activation timing is preserved between the two groups, but the directionality of the activation pattern is inverted.

In Group A the areas presenting the longest LEMD are on the lateroposterior side, whereas in Group B these are on anteroseptal one. LEMD is inhomogeneous in Group B.

The contraction pattern is splitting in two fronts in Group B, favoring posteroseptal base contraction before the apex, which in turn is activating among the firsts in Group A.

The scar burden across Group B is significantly higher in the septal and apex segments, whilst no significant difference is found across Group A.

#### LV segments parameter correlation

To condense the findings and understand the parameter interplay, we have calculated the Pearson’s correlation coefficients for both groups. The heatmaps corresponding to the obtained correlation matrix are showed in Supplementary Fig. S3. The p-values obtained for the correlation between single parameters are in Supplementary Table S6.

**Figure 6:**
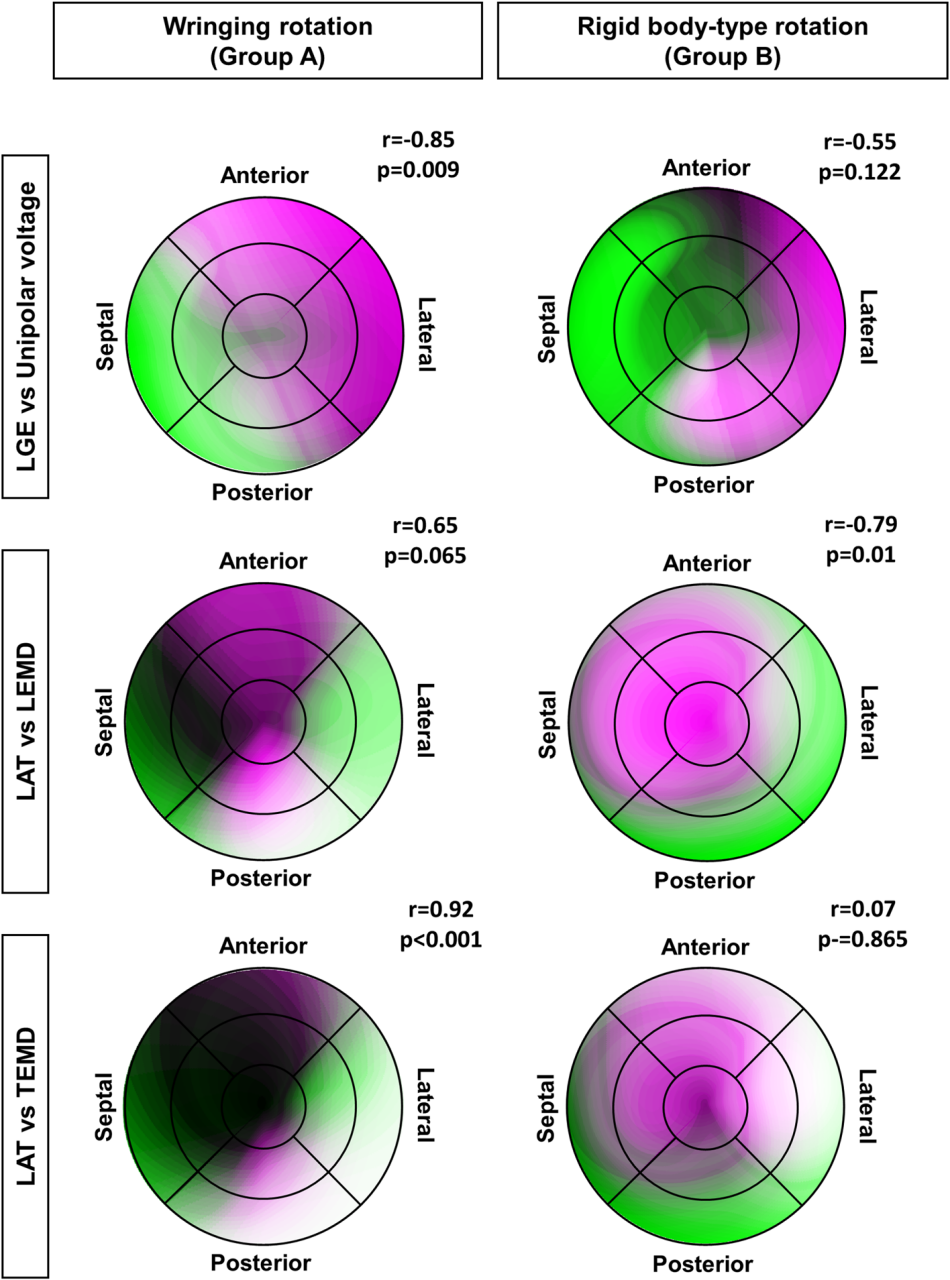
Correlation maps of selected electromechanical parameters. The figures are combination in false colors of the bullseye maps corresponding to the parameters listed in the rows. The higher presence of white and dark areas indicates higher positive correlation.

In Group A, the LGE intensity is highly correlated with areas of low voltage (r = − 0.85, p = 0.009); this correlation is lost in Group B probably due to the voltage increase in the midposterior segment (Fig. 6 top). The LAT and LEMD parameters are inversely correlated (r = − 0.79, p = 0.01) in Group B; this relationship in inverted and close to significance in Group A (Fig. 6 middle). The LAT and the TEMD are strongly correlated in Group A (r = 0.92, p < 0.0001), a correlation not observed in Group B (Fig. 6 bottom). Together these findings summarized in the Box 3.

**Box 3: Summary of LV segments parameter correlation** Scar location is associated with low voltage areas unless electrical changes are underway.

In Group B, with pathological RBT rotation, electrical propagation and EM coupling are uncorrelated.

In Group A, electrical propagation and mechanical activation are highly overlapping, whereas in Group B there is evidence of electromechanical dissociation.

## Discussion

In healthy individuals, electrical signals propagate through the conduction system at a high velocity allowing synchronized yet anisotropic depolarization of left and right ventricle. Furthermore, mechanical function of LV is correlated with several factors: Homogeneous EM coupling; activation of transmural counter-directionally oriented myocardial fibers; epicardially-dominating torque. These factors guarantee a LV wringing motion, which results in clockwise rotation of the base and counterclockwise rotation of the apex^1^.

In a normal adult heart this wringing mechanism plays a pivotal role to achieve LVEF of up to 60% with only 15% myocardial fiber shortening^14^. However, in patients with LBBB, mechanical function of LV is often disturbed, with a heterogeneous conduction pattern presentation, which depends on anatomical location of the block and co-existence of other heart diseases (i.e. coronary artery disease, cardiomyopathies)^15–17^. Interestingly, the global prevalence of isolated LBBB is approximately 1.5%^18^. This form of conduction delay was reported to affect global longitudinal strain in asymptomatic hypertensive patients^19^, and decrease apical rotation thus, consequently, reduce LV twist^20,21^. This was suggested to serve as a biophysical marker of subtle LV dysfunction^21^. LBBB is reported to affect 20–30% of all patients with HF^22^ and recent studies support incorporation of rotational parameters to evaluate LV function in this population^4,23,24^. We have observed in our study that similar manifestation of LBBB can present remarkably different patterns of LV rotation.

Importantly, ischemic HFrEF with concomitant LBBB may result in remodeling of ventricular EM coupling and geometry, disrupting LV mechanical activity and rotation pattern^25–27^. Remarkably, these changes can affect myocardium proximal to the ischemic region, as well as the remote one^28^. Similarly to our findings, Paoletti Perini et al.^4^ demonstrated, that, in patient with HF and co-existing LBBB, reduction of LV apex–basal rotation and global twist was associated with segmental rotational dyssynchrony. Moreover, authors reported the strongest impairment at the apical level confirming previous published outcomes^6,7,29,30^.

In our study, aside from the different rotation pattern which served as sole parameter for grouping, the most striking differences between the two patients’ groups were: A stronger electromechanical correlation in Group A, lost in Group B upon inhomogeneous LEMD and negative correlation with electrical activation; inhomogeneous and higher voltage levels in Group B, which can represent a marker of electrical remodeling; locally higher scar burden in Group B.

LEMD is reported to have a regional gradient from apex to base, as tested in porcine hearts^31^. In that sense, Group B presented the most sever changes in this property. The reason for a different local electro-mechanical delay can be multivariate: β-adrenergic stimulation is drastically deranged during HF, but intriguingly a role of β3 receptors has been shown to compensate β1 de-sensitization, providing sustained inotropic effect (with improvement of EM coupling) and paracrine modulation with benefits in patients with LVEF < 40%. An overregulated adrenergic stimulation could explain also the increased unipolar and bipolar voltage levels observed in RBT rotation group. On the other hand, progressing HF is characterized by cardiomyocyte hypertrophy^32^, which affects calcium signaling^33^ and might explain the regional differences found in RBT group^34^. Together with these biomolecular remodeling, coupling and contraction alterations can derive also at the tissue-level from scar tissue presence, myofibers geometry rearrangement, and right ventricle mechanics under influence of interventricular delays.

Regarding the effect of scar tissue, we did not observe significant differences between the two groups, rather the LGE intensity was similar between the two groups. LGE intensity was correlated with low voltage area when electrical changes are not undergoing (i.e. voltage alterations in Group B). This does not exclude that a non-detected portion of diffuse fibrosis might have determined the LEMD changes.

As of many comparisons in this study, we found an internal heavier burden in the midseptal region of Group B, which is also the region in which the mechanical activation front splits in two, lingering before activating the neighboring apical and anterolateral regions. Maffesanti et al. showed, with a similar setup to the one presented here, how a significant scar burden can significantly alter electromechanical synchronization and activation centroids^28^. This is accordance with our findings, although we could not distinguish between significant and non-significant scar burden, due to small sample size.

**Figure 7:**
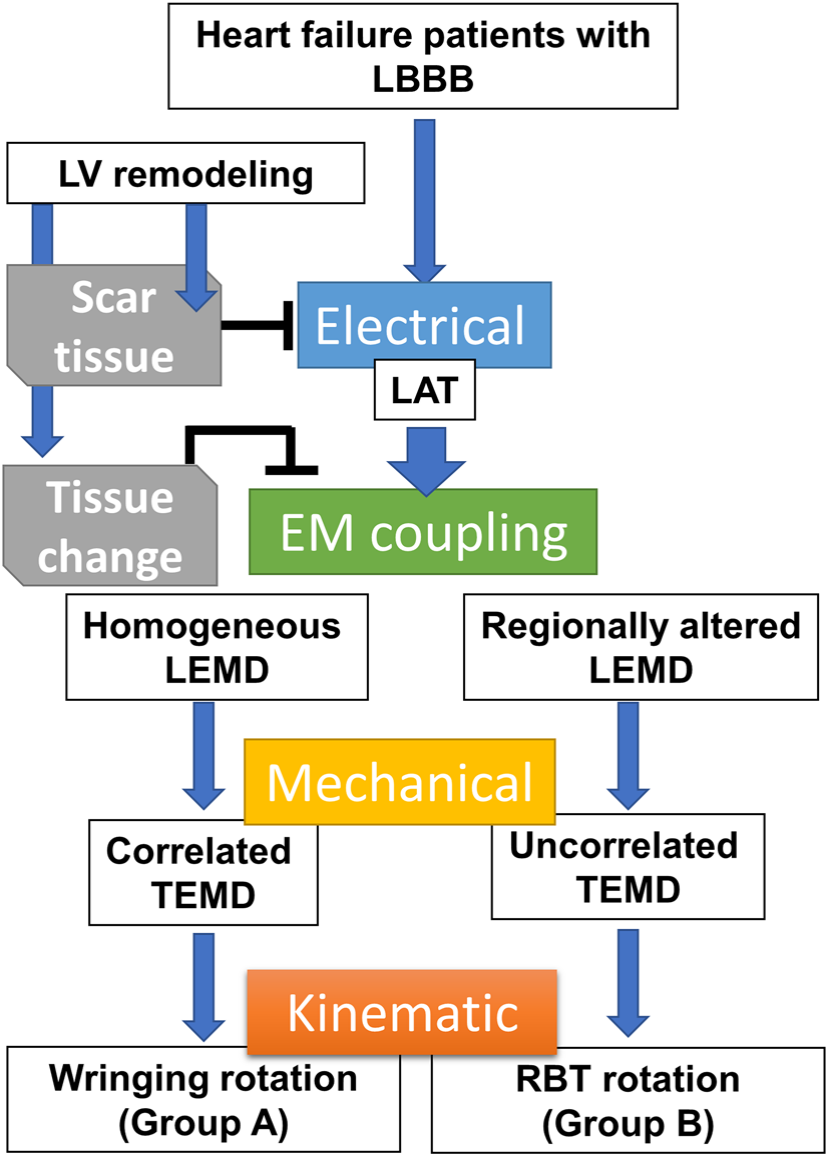
Scheme of the working hypothesis on the origin of differential left ventricle rotational patterns in heart failure patients mapped with an endovascular electromechanical catheter system (NOGA XP). *EM* electro-mechanical, *LAT* local activation time, *LBBB* left bundle branch block, *LEMD* local electro-mechanical delay, *TEMD* total electro-mechanical delay, *RBT* rigid-body-type.

We concluded that electromechanical coupling changes are paramount to determine the LV kinetics, whether these electromechanical changes derive from a substantial scar burden or other underlying remodeling (e.g. electrical) phenomena. A working model of our fions is shown in Fig. 7. Briefly, the presence of an ischemic event causes electrical changes and proximal/remote myocardial EM remodeling. Electrical activation pattern is similar in HF LBBB patients with either preserved LV wringing rotation or RBT rotation. A remarked loss of the physiological gradient in electromechanical coupling causes LV regional delays and a consequent dyssynchronous mechanical activation. Our study also stresses how internal and segmental variability must be taken into consideration to understand the origin of different LV kinetics.

Noteworthy, to provide reliable and high-resolution segmental information about EM properties, we used NOGA XP catheter-based technology for a direct analysis of rotational behavior. In our opinion this methodological approach provides an unique analytical value in comparison to previously applied techniques (tagged magnetic resonance imaging [MRI]^35^, feature tracking MRI^36^, tissue Doppler^37^, and speckle tracking echocardiography^1^). Important methodological advantages of the NOGA XP system include: (1) direct and simultaneous measurement of electrical and mechanical activation at segmental level, (2) utilization of the earliest electrical activation of LV instead of (a) surface ECG or (b) normalized percentage of systolic and diastolic duration based on aortic valve closure, which gives more precise time reference point in case of NOGA XP system. The data presented here serve therefore as a strong basic groundwork: if the working hypothesis is proven clinically predictive of CRT responsiveness, this study could serve as reference for the collection of electromechanical biomarkers—nominally scar tissue burden, electrical activation pattern, mechanical activation and electromechanical coupling—with available combined non-invasive imaging, such as electro-cardiographic imaging and cMR.

## Limitations

The presented results are hypothesis generating and should be considered with a caution due to relatively small number of study participants and other study limitations: (1) NOG-based EM characteristics of LV is dependent on the completeness of the endocardial cavity mapping (however, we obtained 273 ± 47 mapping points per patient with adequate 3D location representing LV geometry), (2) endocardial contact mapping does not provide any information about epicardial mechanical function, (3) LEMD parameter was defined as a time interval between the local electrical activation of the segment and its peak of systolic rotation, not the onset of mechanical activation (this approach is accepted in human studies on LV mechanics^4^ as the onset of segmental mechanical activation is difficult to annotate; instead, peak of systolic rotation can be easily detected providing reliable and comparable results among studies), (4) no healthy control group due to invasiveness of NOGA XP mapping procedure.

## Methods

### Study population

Between April 2015 and May 2017, thirty HFrEF patients were sequentially enrolled in the study before the implantation of resynchronization device.

*Inclusion criteria* (1) Age 18–75 years, (2) Ischemic cardiomyopathy, (3) LVEF < 35%, (4) Functional class NYHA II/III or ambulatory IV, despite at least 30 days of optimal medical treatment, (5) Sinus rhythm, (6) Left Bundle Branch Block (LBBB), (7) QRS duration > 150 ms, (8) Signed written informed consent.

*Exclusion criteria* (1) Coronary artery disease requiring revascularization, (2) Acute coronary syndrome < 3 months prior to study enrolment, (3) Implantation of pacemaker (4) Presence of implantable cardioverter-defibrillator, (5) Left ventricle thrombus or aneurysm, (6) Severe aortic stenosis, (7) Renal failure (GFR < 30 mL/min/1.73 m^2^), (8) History of neoplasm, (9) Bleeding diathesis, (10) HIV, HBV, HCV infection, (11) Pregnancy, (12) Contrast allergy.

The study conforms to the Declaration of Helsinki. It was approved by the Ethics Committee of the Medical University of Silesia in Katowice. All patients have signed the informed consent form.

### Transthoracic echocardiography

Transthoracic echocardiography was performed using cardiac ultrasound machine (Epiq 7G, Philips Ultrasound, Inc., Reedsville, PA, USA) equipped with S5-1 probe (2.5–3.5 MHz) by expert cardiologist according to the American Society of Echocardiography recommendations^38^. Sector width and gain settings were adjusted for grayscale 2D imaging with second harmonic mode activated. From the complete echocardiographic report, the following parameters were evaluated: left ventricle end-diastolic volume (LVEDV) and left ventricle end-systolic volume (LVESV) and LVEF based on the Simpson method.

### Late gadolinium—enhanced cardiac MRI

Cardiovascular magnetic resonance imaging was performed as described by Maffesanti et al.^28^ Briefly, patients (5 for Group A and 20 for Group B) were scanned using a 1.5 T scanner (SIGNA, GE Medical Systems, USA) equipped with standard torso coil. Short-axis late gadolinium enhancement (LGE) images were obtained 7–12 min after the intravenous bolus injection of gadolinium (0.2 mmol/kg body weight). Scans were subsequently analyzed via CVI42 v.5.11.2. Each short-axis slice was subdivided into the 16 angular sectors, according to the AHA standard and the local scar burden was expressed as the percent of grey area per subregion. The resulting colormaps were converted into nine segments values by weighted mean based on segment area. The nine segment colormaps were post-processed as described below.

### Electro-mechanical mapping

The 3D electro-mechanical mapping system NOGA XP (Biosense Webster, Inc., Irvine, CA, USA) allows simultaneous measurement of local electrical activity and mechanical motion of myocardial tissue. The data are generated by electro-magnetic tracking of a miniaturized sensor located at the tip of the mapping catheter (NOGAStar, Biosense Webster Inc., Irvine, CA, USA), as described in detail previously^39^. Internal standardized NOGA-algorithms identified and eliminated points with unstable wall contact and inappropriate rhythm or wall movement.

### Post-processing of LV rotational and electro-mechanical data

After filtering of the mapping points and assessment of an adequate catheter-endocardium contact, data were exported for analysis of rotation angles using external software algorithms. Rotational parameters were calculated by recording angular displacement of the individual mapping points around the heart axis, defined by the geocenter of all mapping points and the apex of the heart. The position of each mapping point was recorded in three dimensions at 10 ms intervals over a period of three heart cycles. Rotational values were calculated between LV end-diastolic to end-systolic phase. Similarly, local activation time (LAT), local rotational electro-mechanical delay (LEMD) and total rotational electro-mechanical delay (TEMD) were computed as shown in Fig. 1a. Acquired mapping points were positioned on the two-dimensional 9-segment model of the LV (bulls-eye projection, Fig. 1b) according to the corresponding tridimensional point coordinates. We divided the LV bulls-eye projection in four basal segments (basoseptal, basolateral, posterobasal, anterobasal), four mid-ventricular (midseptal, midlateral, midposterior, midanterior), and one apical segment. Each segment contains an averaged value of the parameter of interest in that position. These nine segments values were further aggregated in three sectors (apex, medial and basal sector) or across the whole LV to allow multilevel analysis (see Supplementary Fig. S1 online). When viewed in projection from the LV apex, clockwise rotation was labeled as a negative value, whereas a counterclockwise rotation as a positive value.

After processing, rotation data were also transferred into a NOGA 3D viewer and visualized as color-coded maps (Fig. 2, clockwise rotation < − 3° expressed in red color; counterclockwise rotation > 3° expressed in purple color).

High resolution bullseye plots were obtained by oversampling segmental values by a factor 100, then these were interpolated with spline method and plotted in a polar graph by a custom script in Matlab R2017a (MathWorks, Natick, MA, USA), as shown in Fig. 3.

### List of global, sectorial, and segmental parameters

A complete description of the computed and analyzed parameters is provided in Supplementary Table S1 online. Briefly, the following parameters were used to assess LV global (G), sectorial (SEC) and segmental (SEG) rotational EM characteristics, based on the level of analysis: (1) LV peak torsion—G; (2) LV mean torsion—G; (3) Rotation angle—G; SEC, SEG; (4) Rotation rate—G, SEC, SEG; (5) Interventricular delay—G; (6) LV electrical activation time—G; (7) LV electrical cycle length—G; (8) LV mechanical cycle length—G; (9) Unipolar voltage—G, SEC, SEG; (10) Bipolar voltage—G, SEC, SEG; (11) LAT—SEG, SEC; (12) LEMD – SEC, SEG; (13) LV rotational electro-mechanical delay – G; (14) TEMD – SEC, SEG.

### Statistical analysis

Values are presented as the mean ± standard deviation (SD) or median (25th; 75th percentile), according to data distribution. Normality was verified by Shapiro–Wilk test. Qualitative data are expressed as crude values and/or percentages. Difference between global parameters were analyzed by non-paired t-test (if necessary, with Welch’s correction) for normally distributed data or Mann–Whitney’s test for nonnormally distributed data. Differences between sectorial and segmental groups were analyzed using 2-way analysis of variance (ANOVA). Multiple comparisons within each group were corrected via Holm-Sidak’s method. To reduce the number of comparisons among segments in the intragroup analysis, we first computed all the possible pairs and chose as control the most repeated segment with lowest p-values. Statistical analysis and graphs were performed using MedCalc version 19.0.7 (MedCalc Software bvba, Ostend, Belgium) and Prism 8 (GraphPad, La Jolla, CA, USA) and the significance level was p ≤ 0.05).

### Ethical statement

No animal studies were carried out by the authors for this article.

## Supplementary Information

Supplementary Information 1.

Supplementary Video S1.

Supplementary Video S2.

Supplementary Video S3.

## Abbreviations

3D: Three-dimensional
HF: Heart failure
HFrEF: Heart failure with reduced ejection fraction
LBBB: Left bundle branch block
LGE: Late gadolinium enhancement
LV: Left ventricle
LVEF: LV ejection fraction
MRI: Magnetic resonance imaging
RBT: Rigid body-type
EM: Electro-mechanical
LEMD: Local rotational electro-mechanical delay
TEMD: Total rotational electro-mechanical delay
