# Supplementary material for: Local electromechanical alterations determine the left ventricle rotational dynamics in CRT-eligible heart failure patients
Source: Sci Rep. 2021 Feb 5;11:3267. doi: 10.1038/s41598-021-82793-1 (PMC7865069; doi:10.1038/s41598-021-82793-1)
Supplement: Supplementary file 1 — Supplementary Information 1. [file 41598_2021_82793_MOESM1_ESM.pdf]

Title: Local electromechanical alterations determine the left ventricle rotational dynamics in CRT-eligible heart failure patients

Supplementary materials

Authors: Tomasz Jadczyk[1,2#], Radoslaw Kurzelowski[1#], Krzysztof S. Golba[3], Jacek Wilczek[3], Guido Caluori[2,4], Francesco Maffessanti[5], Jolanta Biernat[3], Katarzyna Gruszczynska[6], Magdalena Cybulska[3], Maximilian Y. Emmert[7,8], Zofia Parma[1], Kamil Baranski[9], Mieczyslaw Dutka[10], Barbara Kalanska[1], Zdenek Starek[2,11] and Wojciech Wojakowski[1]*

[#]equal contribution

Affiliations:

[1]Department of Cardiology and Structural Heart Disease, Medical University of Silesia, Katowice, Poland

[2]Interventional Cardiac Electrophysiology Group, International Clinical Research Center, St. Anne's University Hospital Brno, Czech Republic

[3]Department of Electrocardiology and Heart Failure, Medical University of Silesia, Katowice, Poland

[4]IHU-LIRYC, Inserm U1045 (CRBCT), Bordeaux, France

[5]Center for Computational Medicine in Cardiology, Università della Svizzera Italiana, Lugano, Switzerland

[6]Department of Diagnostic Imaging, Medical University of Silesia, Katowice, Poland.

[7]Department of Cardiovascular Surgery, Charité Universitätsmedizin Berlin, Berlin, Germany

[8]Department of Cardiothoracic and Vascular Surgery, German Heart Center Berlin, Berlin, Germany

[9]Department of Epidemiology, Medical University of Silesia, Katowice, Poland

[10]Department of Biochemistry and Molecular Biology, Faculty of Health Sciences, University of Bielsko-Biala, Bielsko-Biała, Poland

[11]1st Department of Internal Medicine - Cardioangiology, St. Anne's University Hospital Brno, Czech Republic

Address for correspondence

Wojciech Wojakowski MD, PhD

Department of Cardiology and Structural Heart Diseases

Medical University of Silesia

Ziołowa 45-47, Katowice, Poland

Tel: +48-32 2523930, Fax: +48-32 2523930

e-mail: wwojakowski@sum.edu.pl

1. Supplementary Figures

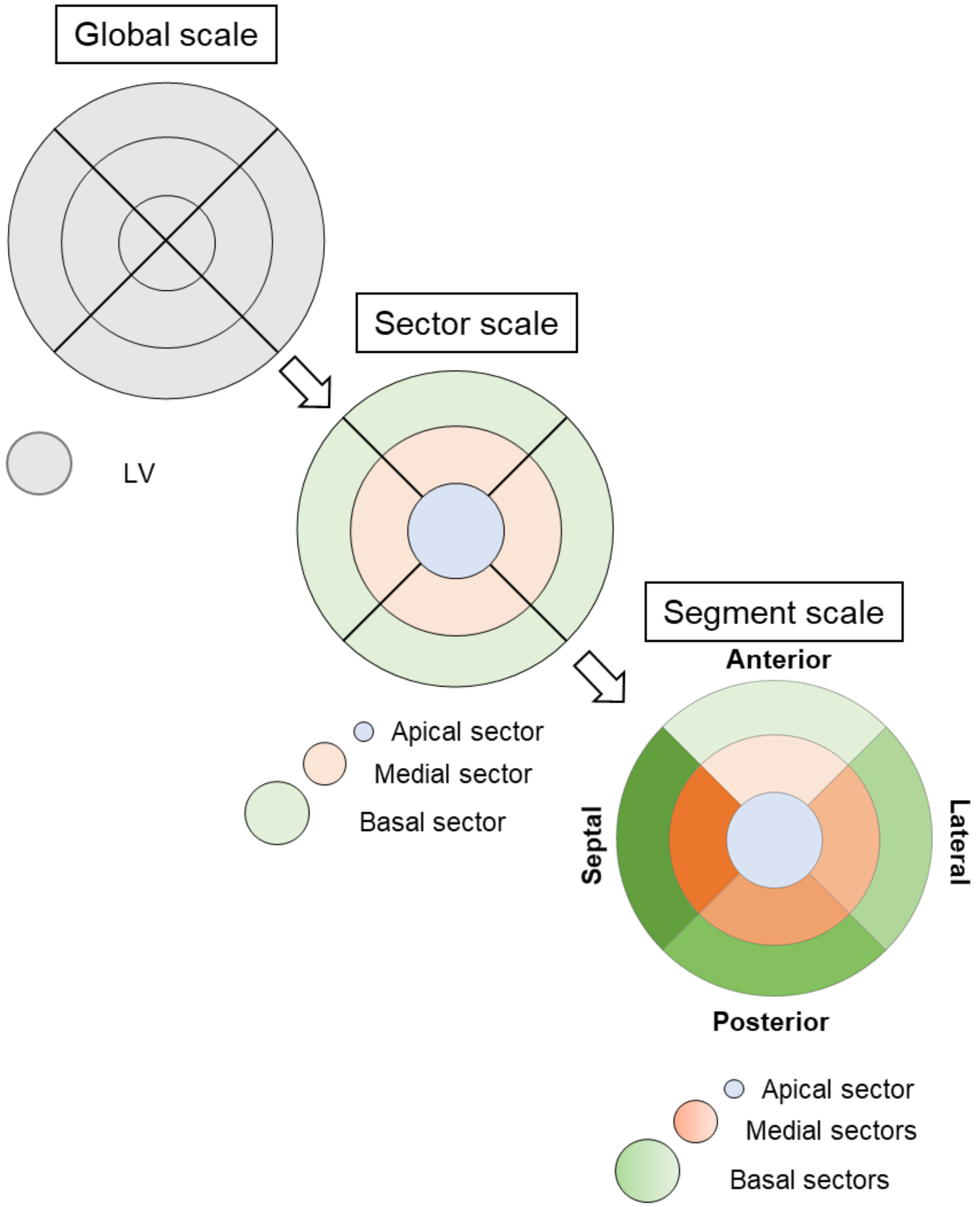

Supplementary Figure S1. Schematic representation of the multiscale approach for the parameter analysis used in this study

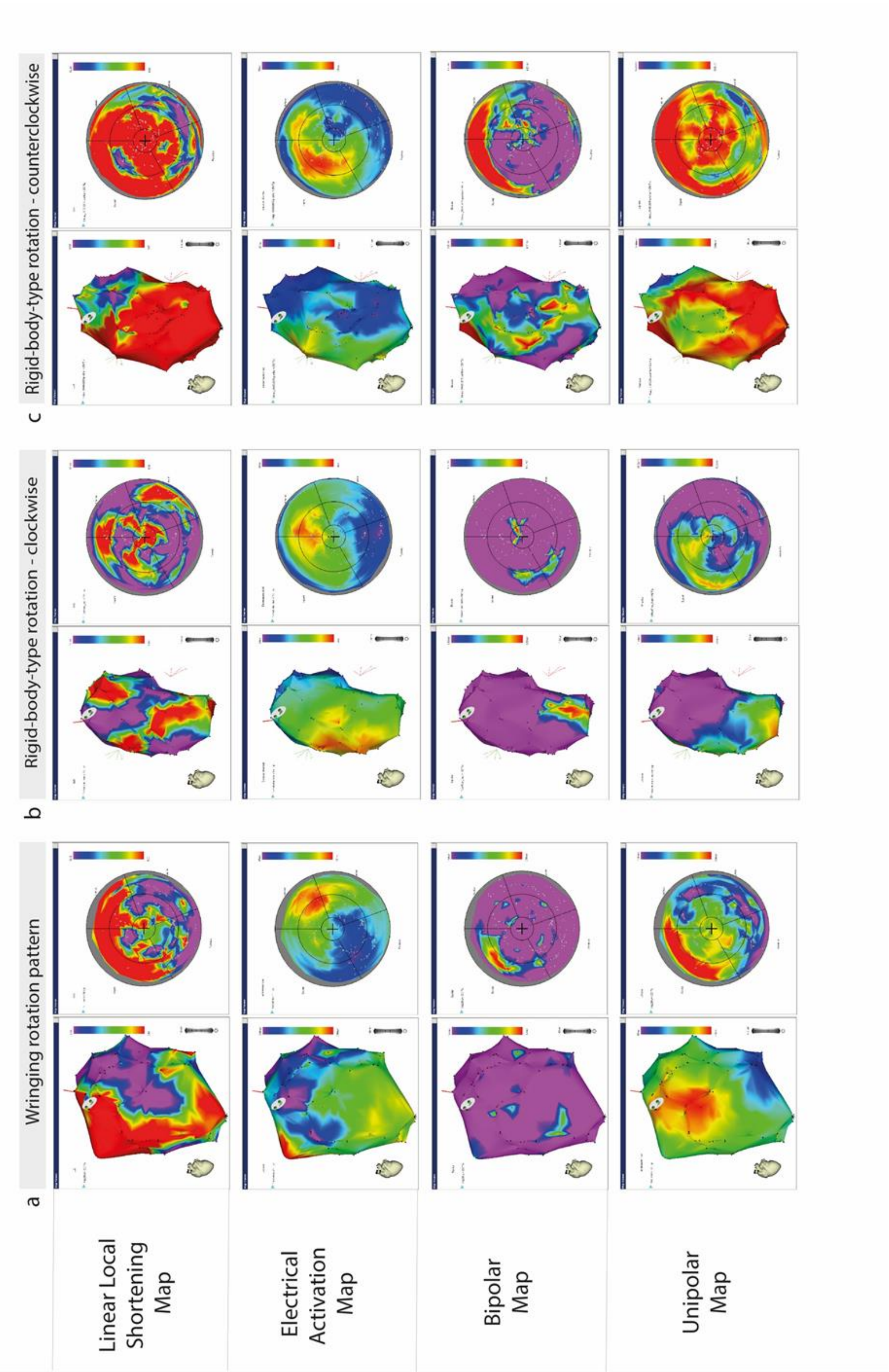

Supplementary Figure S2. Representative 3D and bullseye maps of cardiac parameters of the LV endocardium detected with the employed electromechanical system.

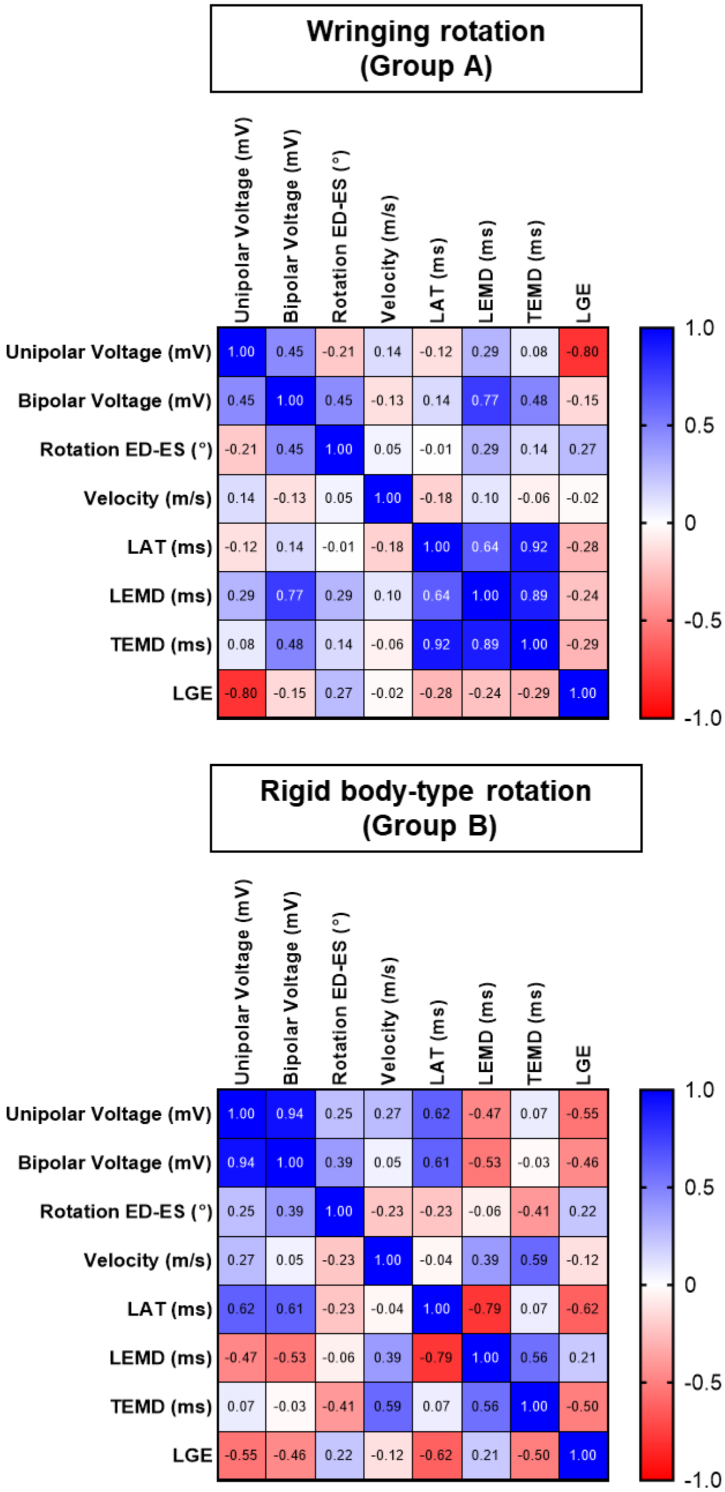

Supplementary Figure S3. Correlation matrix of the Pearson's' coefficients for wringing rotation (Group A, top) and rigid-body-type rotation (Group B, bottom)

2. **Supplementary tables**

Supplementary Table S1 Basic definitions and parameters used to assess LV electro-mechanics

**Parameter (unit)**	**Definition**	**Level of analysis (Global=G, Sectorial = SEC, Segmental=SEG)**
LV peak torsion (°)	Maximum apex–base (maximum value among 4 basal segments) circumferential-longitudinal shear angle of the LV; according to the formula by Russel et al.[1]<br>$$T = \frac{(\phi_{apex}\rho_{apex} - \phi_{base}\rho_{base})}{D}$$<br>T – torsion<br>⌀apex – rotation of the apex<br>⌀base – rotation of the base<br>ρ apex - radius of the apex<br>ρ base - radius of the base<br>D – distance between the apex and the base	G
LV mean torsion (°)	Mean apex-base (mean value of 4 basal segments) circumferential-longitudinal shear angle of the LV; according to the formula by Russel et al. [1].	G
Rotation angle (°)	Mean systolic rotation of the each segment or	SEC, SEG

	sector short-axis cross section as viewed from the apex	
Rotation rate (°/s)	Mean value of systolic angular displacement rate	G, SEC, SEG
Interventricular delay (ms)	Delay between electrical activation of the right and left ventricle - measured as time delay from ECG lead V1 to earliest LV endocardial electrical activation registered at the mapping catheter	G
LV electrical activation time (ms)	Time between the earliest and the latest LV electrical activation	G
LV electrical cycle length (ms)	ECG interval between two successive R wave peaks	G
LV mechanical cycle length (ms)	Interval between two peak systolic mechanical activations	G
Unipolar voltage (mV)	Endocardial voltage measured as unipolar amplitude at the tip-electrode of the catheter	G, SEC, SEG
Bipolar voltage (mV)	Endocardial voltage measured as bipolar amplitude between tip- and ring-electrode of the catheter	G, SEC, SEG
Local activation time, LAT (ms)	Time interval between earliest electrical activation in LV and local electrical activation in the respective segment	SEC, SEG
Local rotational electro-	Time interval between the local electrical	SEC, SEG

mechanical delay, LEMD (ms)	activation of the segment and its local systolic peak rotation	
LV rotational electro-mechanical delay (ms)	Average 9 segments LEMD	G
Total rotational electro-mechanical delay, TEMD (ms)	Time interval between earliest electrical activation of the LV and systolic rotation peak of the segment	G, SEC, SEG
Late gadolinium enhancement, LGE, intensity (%)	Percentage of the segment area with intensified brighter signal	G, SEC, SEG

LAT, Local activation time. LGE, Late gadolinium enhancement. LEMD, Local rotational electro-mechanical delay. LV, Left ventricle TEMD, Total rotational electro-mechanical delay.

Supplementary Table S2 Sectorial EM comparison between LV with preserved rotation and rigid body-type rotation

Parameter	Sector	Group A	Group B	P value
Unipolar voltage (mV)	Apical	7.31 ± 2.13	8.33 ± 2.87	0.683
	Medial	8.14 ± 0.73	10.06 ± 2.21	0.175
	Basal	7.72 ± 2.12	10.03 ± 1.70	0.075
Bipolar voltage (mV)	Apical	2.28 ± 1.18	2.0 ± 0.76	0.898
	Medial	2.29 ± 0.84	2.89 ± 1.16	0.455
	Basal	1.91 ± 0.68	3.02 ± 1.01	0.044
Rotation angle (°)	Apical	7.0 ± 3.8	-5.5 ± 6.0	<0.0001
	Medial	1.0 ± 2.6	-4.5 ± 1.4	0.003
	Basal	-2.2 ± 2.2	-6.4 ± 1.6	0.035
Rotation velocity (m/s)	Apical	48.3 ± 30.6	40.2 ± 15.3	0.343
	Medial	23.25 ± 0.6	41.13 ± 2.7	0.003
	Basal	31.50 ± 8.3	38.7 ± 5.5	0.443
LAT (ms)	Apical	26 ± 15	14 ± 14	0.183
	Medial	34 ± 21	26 ± 13	0.498
	Basal	53 ± 13	46 ± 10	0.597
LEMD (ms)	Apical	373 ± 72	383 ± 56	0.897
	Medial	362 ± 20	379 ± 12	0.682
	Basal	375 ± 13	349 ± 14	0.302
TEMD (ms)	Apical	398 ± 70	397 ± 53	0.999
	Medial	396 ± 39	100 ± 16	0.989

	Basal	429 ± 22	395 ± 14	0.128
LGE Intensity (%)	Apical	13.33±10.27	11.19±3.29	0.7006
	Medial	3.9±2.91	6.96±4.68	0.4200
	Basal	3.91±4.05	5.83±3.04	0.7649

LAT, Local activation time. LGE, Late gadolinium enhancement. LEMD, Local rotational electro-mechanical delay. TEMD, Total rotational electro-mechanical delay. Values are mean ± SD

Supplementary Table S3 Sectorial EM comparison within groups with wringing rotation (Group A) and rigid-body-type rotation (Group B)

Parameter	Comparison	P value (Group A)	P value (Group B)
Unipolar voltage (mV)	Apical vs Medial	0.889	0.025
	Apical vs Basal	0.985	0.029
	Basal vs Medial	0.983	>1.000
Bipolar voltage (mV)	Apical vs Medial	>0.9999	0.007
	Apical vs Basal	0.885	0.002
	Basal vs Medial	0.877	0.956
Rotation angle (°)	Apical vs Medial	0.016	0.731
	Apical vs Basal	<0.0001	0.756
	Basal vs Medial	0.310	0.200
Rotation velocity (m/s)	Apical vs Medial	0.001	0.990
	Apical vs Basal	0.042	0.959
	Basal vs Medial	0.527	0.851
LAT (ms)	Apical vs Medial	0.685	0.013
	Apical vs Basal	0.002	<0.0001
	Basal vs Medial	0.037	<0.0001
LEMD (ms)	Apical vs Medial	0.943	0.967
	Apical vs Basal	0.999	0.004
	Basal vs Medial	0.900	0.015
TEMD (ms)	Apical vs Medial	0.999	0.987
	Apical vs Basal	0.385	0.996

	Basal vs Medial	0.311	0.944
LGE Intensity (%)	Apical vs Medial	0.003	0.009
	Apical vs Basal	0.003	0.0007
	Basal vs Medial	>0.999	0.801

LAT, Local activation time. LGE, Late gadolinium enhancement. LEMD, Local rotational electro-mechanical delay. TEMD, Total rotational electro-mechanical delay. Values are mean ± SD

Supplementary Table S4 Segmental EM comparison between groups with wringing rotation (Group A) and rigid-body-type rotation (Group B)

Parameter	Segment	Group A	Group B	P value
Unipolar voltage (mV)	Apex	7.31 ± 2.13	8.54 ± 2.87	0.999
	Midanterior	9.13 ± 1.61	7.93 ± 3.79	>1.000
	Anterobasal	10.43 ± 2.28	8.78 ± 3.02	1.000
	Midlateral	7.89 ± 1.80	9.97 ± 3.87	0.554
	Basolateral	8.65 ± 3.00	12.07 ± 4.74	0.247
	Midposterior	8.39 ± 3.92	12.45 ± 4.68	0.023
	Posterobasal	7.16 ± 3.36	10.00 ± 4.04	0.216
	Midseptal	7.14 ± 1.93	6.76 ± 3.04	>1.000
	Basoseptal	4.65 ± 2.09	7.72 ± 3.01	0.520
Bipolar voltage (mV)	Apex	2.28 ± 1.18	2 ± 0.76	>1.000
	Midanterior	1.61 ± 0.9	1.85 ± 1.3	>1.000
	Anterobasal	2.37 ± 2.45	2.51 ± 1.64	>1.000
	Midlateral	1.84 ± 0.63	3.06 ± 2.11	0.780
	Basolateral	1.91 ± 1.17	4.06 ± 2.35	0.107
	Midposterior	3.73 ± 3.49	4.71 ± 2.33	0.927
	Posterobasal	2.56 ± 2.08	3.9 ± 2.82	0.678
	Midseptal	1.97 ± 1.51	1.92 ± 1.48	>1.000
	Basoseptal	0.8 ± 0.69	1.62 ± 1.25	0.976
Rotation angle (°)	Apex	7.0 ± 3.8	-5.5 ± 6.0	<0.0001
	Midanterior	-2.4 ± 3.3	-6 ± 4.8	0.745

	Anterobasal	-3.6 ± 5.7	-7.9 ± 6.1	0.515
	Midlateral	3.6 ± 3.9	-4.7 ± 6.2	0.007
	Basolateral	-4.2 ± 2.4	-7.7 ± 5.3	0.748
	Midposterior	3.5 ± 4.1	-2.2 ± 6.4	0.156
	Posterobasal	1.4 ± 1.7	-3.9 ± 3.8	0.229
	Midseptal	-0.4 ± 3.1	-5.1 ± 4.7	0.380
	Basoseptal	-2.4 ± 4.2	-6.0 ± 6.4	0.745
Rotation velocity (m/s)	Apex	48.3 ± 30.6	40.0 ± 15.3	0.945
	Midanterior	23.2 ± 6.9	37.2 ± 12.5	0.425
	Anterobasal	40.8 ± 25.0	41.2 ± 16.8	>1.000
	Midlateral	23.1 ± 12.1	44.3 ± 16.9	0.041
	Basolateral	35.6 ± 16.1	44.0 ± 15.9	0.932
	Midposterior	22.5 ± 8.8	42.7 ± 22.4	0.059
	Posterobasal	18.3 ± 12.1	29.4 ± 12.4	0.728
	Midseptal	24.1 ± 9.3	40.3 ± 13.5	0.236
	Basoseptal	31.3 ± 9.8	40.1 ± 17.3	0.906
LAT (ms)	Apex	26 ± 15	14 ± 14	0.770
	Midanterior	14 ± 12	19 ± 20	0.999
	Anterobasal	35 ± 19	40 ± 27	0.998
	Midlateral	59 ± 14	46 ± 13	0.581
	Basolateral	67 ± 23	60 ± 17	0.986
	Midposterior	49 ± 24	28 ± 15	0.083
	Posterobasal	65 ± 25	53 ± 13	0.652

	Midseptal	12 ± 19	10 ± 11	>1.000
	Basoseptal	47 ± 25	34 ± 17	0.591
LEMD (ms)	Apex	373 ± 72	383 ± 56	>1.000
	Midanterior	343 ± 74	387 ± 54	0.421
	Anterobasal	381 ± 57	369 ± 44	1.000
	Midlateral	366 ± 40	373 ± 41	>1.000
	Basolateral	377 ± 51	349 ± 37	0.909
	Midposterior	394 ± 41	363 ± 60	0.850
	Posterobasal	389 ± 38	329 ± 45	0.101
	Midseptal	347 ± 46	393 ± 42	0.378
	Basoseptal	353 ± 71	347 ± 65	>1.000
TEMD (ms)	Apex	399 ± 70	397 ± 52	>1.000
	Midanterior	357 ± 74	407 ± 53	0.392
	Anterobasal	416 ± 47	409 ± 49	>1.000
	Midlateral	425 ± 35	418 ± 43	>1.000
	Basolateral	444 ± 43	409 ± 37	0.817
	Midposterior	443 ± 22	375 ± 95	0.073
	Posterobasal	454 ± 14	382 ± 51	0.045
	Midseptal	359 ± 55	402 ± 44	0.566
	Basoseptal	400 ± 52	381 ± 70	0.996
LGE intensity (%)	Apex	13.33±10.27	11.19±10.28	>0.9999
	Midanterior	3.9±2.91	3.93±4.08	>0.9999
	Anterobasal	3.91±4.05	2.5±3.01	>0.9999

	Midlateral	3.44±4.79	3.08±7.94	>0.9999
	Basolateral	2.22±1.22	4±9.53	>0.9999
	Midposterior	9.86±7.58	5.97±7.6	0.9905
	Posterobasal	11.28±9.12	6.22±8.45	0.9465
	Midseptal	17.22±11.69	14.87±16.34	0.9998
	Basoseptal	18.01±12.65	10.58±11.65	0.6549

LAT, Local activation time. LGE, Late gadolinium enhancement. LEMD, Local rotational electro-mechanical delay. TEMD, Total rotational electro-mechanical delay. Values are mean ± SD or median (25th;75th percentile)

Supplementary Table S5 Segmental EM comparison within groups with wringing rotation (Group A) and rigid-body-type rotation (Group B)

Parameter	Comparison	P value (Group A)	P value (Group B)
Unipolar voltage (mV)	Apex vs. Midanterior	0.977	1.000
	Apex vs. Anterobasal	0.672	0.820
	Apex vs. Midlateral	>1.000	0.165
	Apex vs. Basolateral	0.997	**0.002**
	Apex vs. Midposterior	0.999	**<0.0001**
	Apex vs. Posterobasal	>1.000	0.134
	Apex vs. Midseptal	>1.000	0.985
	Apex vs. Basoseptal	0.826	0.989
Bipolar voltage (mV)	Apex vs. Midanterior	0.998	>1.000
	Apex vs. Anterobasal	>1.000	0.967
	Apex vs. Midlateral	>1.000	0.342
	Apex vs. Basolateral	>1.000	**0.001**
	Apex vs. Midposterior	0.798	**<0.0001**
	Apex vs. Posterobasal	>1.000	**0.004**
	Apex vs. Midseptal	>1.000	>1.000
	Apex vs. Basoseptal	0.780	0.995
Rotation angle (°)	Apex vs. Midanterior	**0.020**	>1.000
	Apex vs. Anterobasal	**0.005**	0.616
	Apex vs. Midlateral	0.917	1.000

	Apex vs. Basolateral	**0.003**	0.710
	Apex vs. Midposterior	0.910	0.252
	Apex vs. Posterobasal	0.445	0.952
	Apex vs. Midseptal	0.131	>1.000
	Apex vs. Basoseptal	**0.019**	>1.000
Rotation velocity (m/s)	Apex vs. Midanterior	0.061	0.997
	Apex vs. Anterobasal	0.988	>1.000
	Apex vs. Midlateral	0.060	0.980
	Apex vs. Basolateral	0.790	0.986
	Apex vs. Midposterior	**0.049**	0.999
	Apex vs. Posterobasal	**0.012**	0.165
	Apex vs. Midseptal	0.080	>1.000
	Apex vs. Basoseptal	0.440	>1.000
LAT (ms)	Apex vs. Midanterior	0.902	0.957
	Apex vs. Anterobasal	0.979	**<0.0001**
	Apex vs. Midlateral	**0.009**	**<0.0001**
	Apex vs. Basolateral	**0.001**	**<0.0001**
	Apex vs. Midposterior	0.166	0.051
	Apex vs. Posterobasal	**0.001**	**<0.0001**
	Apex vs. Midseptal	0.788	0.981
	Apex vs. Basoseptal	0.250	**0.001**
LEMD (ms)	Apex vs. Midanterior	0.951	>1.000
	Apex vs. Anterobasal	>1.000	0.964

	Apex vs. Midlateral	>1.000	0.995
	Apex vs. Basolateral	>1.000	0.177
	Apex vs. Midposterior	0.995	0.777
	Apex vs. Posterobasal	0.999	**0.003**
	Apex vs. Midseptal	0.978	0.998
	Apex vs. Basoseptal	0.996	0.121
TEMD (ms)	Apex vs. Midanterior	0.832	0.999
	Apex vs. Anterobasal	0.999	0.994
	Apex vs. Midlateral	0.986	0.825
	Apex vs. Basolateral	0.749	0.993
	Apex vs. Midposterior	0.784	0.756
	Apex vs. Posterobasal	0.514	0.965
	Apex vs. Midseptal	0.861	>1.000
	Apex vs. Basoseptal	>1.000	0.949
LGE intensity (%)	Midseptal vs. Apex	0.9966	0.8515
	Midseptal vs. Midanterior	0.1802	**0.0020**
	Midseptal vs. Anterobasal	0.1809	**0.0003**
	Midseptal vs. Midlateral	0.1496	**0.0007**
	Midseptal vs. Basolateral	0.0881	**0.0022**
	Midseptal vs. Midposterior	0.8515	**0.0220**
	Midseptal vs. Posterobasal	0.9507	**0.0287**
	Midseptal vs. Basoseptal	>0.9999	0.7172

LAT, Local activation time. LEMD, Local rotational electro-mechanical delay. TEMD, Total rotational electro-mechanical delay. Values are mean ± SD or median (25th;75th percentile)

Supplementary Table S6: P values of the of the Pearson's' coefficients for wringing rotation (Group A) and rigid-body-type rotation (Group B)

Wringing rotation (Group A)								
	Unipolar Voltage (mV)	Bipolar Voltage (mV)	Rotation ED-ES (°)	Velocity (m/s)	LAT (ms)	LEMD (ms)	TEMD (ms)	LGE
Unipolar Voltage (mV)		0.219	0.583	0.726	0.754	0.444	0.839	**0.009**
Bipolar Voltage (mV)	0.219		0.226	0.748	0.715	0.016	0.193	0.699
Rotation ED-ES (°)	0.583	0.226		0.905	0.985	0.457	0.714	0.490
Velocity (m/s)	0.726	0.748	0.905		0.637	0.804	0.882	0.956
LAT (ms)	0.754	0.715	0.985	0.637		0.065	**0.0005**	0.457
LEMD (ms)	0.444	0.016	0.457	0.804	0.065		**0.001**	0.535
TEMD (ms)	0.839	0.193	0.714	0.882	**0.0005**	**0.001**		0.447
LGE	**0.009**	0.699	0.490	0.956	0.457	0.535	0.447	

Rigid-body-type rotation (Group B)								
	Unipolar Voltage (mV)	Bipolar Voltage (mV)	Rotation ED-ES (°)	Velocity (m/s)	LAT (ms)	LEMD (ms)	TEMD (ms)	LGE
Unipolar Voltage (mV)		0.0002	0.521	0.489	0.074	0.199	0.851	0.127
Bipolar Voltage (mV)	0.0002		0.299	0.894	0.079	0.145	0.945	0.213
Rotation ED-ES (°)	0.521	0.299		0.559	0.555	0.879	0.277	0.565
Velocity (m/s)	0.489	0.894	0.559		0.922	0.297	0.097	0.755
LAT (ms)	0.074	0.079	0.555	0.922		**0.011**	0.864	0.073
LEMD (ms)	0.199	0.145	0.879	0.297	**0.011**		0.119	0.586
TEMD (ms)	0.851	0.945	0.277	0.097	0.865	0.119		0.169
LGE	0.127	0.213	0.565	0.755	0.073	0.586	0.169	

Reference:

1 Russel, I. K., Tecelao, S. R., Kuijer, J. P., Heethaar, R. M. & Marcus, J. T. Comparison of 2D and 3D calculation of left ventricular torsion as circumferential-longitudinal shear angle using cardiovascular magnetic resonance tagging. *J Cardiovasc Magn Reson* **11**, 8 (2009).
